# Regeneration of Peripheral Blood T-Cell Subpopulations in Children After Completion of Acute Lymphoblastic Leukemia Treatment

**DOI:** 10.3390/ijms262211107

**Published:** 2025-11-17

**Authors:** Bartosz Perkowski, Łukasz Słota, Aleksandra Lasia, Tomasz Szczepański, Łukasz Sędek

**Affiliations:** 1 Department of Pediatric Hematology and Oncology, Medical University of Silesia in Katowice, ul. 3 Maja 13-15, 41-800 Zabrze, Poland; bartosz.perkowski@sum.edu.pl (B.P.); lslota@sum.edu.pl (Ł.S.); aleksandra.lasia@sum.edu.pl (A.L.); tszczepanski@sum.edu.pl (T.S.); 2 Department of Microbiology and Immunology, Medical University of Silesia in Katowice, ul. Jordana 19, 41-808 Zabrze, Poland

**Keywords:** flow cytometry, acute lymphoblastic leukemia, immune reconstitution, T-cell subpopulation, chemotherapy, pediatric oncology

## Abstract

Childhood acute lymphoblastic leukemia (ALL) is the most common pediatric cancer, and while chemotherapy has significantly improved survival rates, it can also lead to long-term side effects, including immune system dysfunction. This study aimed to investigate in detail, using flow cytometry, the T-cell subpopulations in the peripheral blood of children who have completed ALL treatment and compare them to a group of healthy children. The study group consisted of 20 patients, aged 5 to 18 years, with blood samples collected at least one year after treatment completion. Of the 52 T-cell subpopulations analyzed, 16 showed statistically significant differences. Children after ALL treatment had higher absolute values of TCRγδ+, double-positive CD4+CD8+ and CD8+ T cells. They also had higher absolute numbers of memory T cells, including total CD45RO+ T cells, and the CD45RO+CD8+ and CD45RO+CD27+ subpopulations. Furthermore, post treatment patients showed higher absolute values of activated T cells (HLA-DR+, HLA-DR+CD8+, HLA-DR+CD57+, and CD25+CD8+), as well as CD57+ and CCR7+ T cells. The absolute leukocyte and granulocyte counts were lower in the study group, while the total lymphocyte count was significantly higher compared to the control group. The findings indicate persistent changes in T-cell subpopulations after ALL treatment, suggesting ongoing immune system rebuilding and chronic antigenic stimulation, possibly due to viral reactivation or chemotherapy-related tissue damage. The increased number of TCRγδ+ cells, which are responsible for eliminating cancer cells, may be a positive aspect of this rebuilding.

## 1. Introduction

Childhood acute lymphoblastic leukemia (ALL) is a heterogeneous hematological malignancy characterized by the uncontrolled proliferation of immature lymphoid cells in the bone marrow (BM) and their possible occurrence in peripheral blood (PB). It is the most common cancer type in children, accounting for approximately 25% of all pediatric malignancies and representing a significant cause of morbidity and mortality in this population [1,2].

The etiology of childhood ALL is complex and multifactorial, involving a combination of genetic predispositions and environmental exposures [3]. Advances in genomic and transcriptomic profiling have unveiled a diverse landscape of genetic alterations that drive leukemogenesis, leading to a better understanding of ALL subtypes and their diversified prognosis and treatment response [4]. These discoveries have paved the way for the development of risk-stratified treatment protocols and targeted therapies, aiming to minimize toxicity while maximizing efficacy.

While substantial progress in treatment strategies over the past few decades has dramatically improved survival rates, with cure rates now exceeding 90% for standard-risk ALL, some patients still face challenges such as treatment resistance, relapse, and long-term side effects from intensive chemotherapy [5,6]. A subset of patients, particularly those with high-risk genetic features or relapsed disease, continues to have poor outcomes. Furthermore, the long-term sequelae of treatment, including secondary malignancies, cardiovascular complications, and neurocognitive impairments, highlight the need for less toxic and more precise therapeutic approaches [7,8]. Another consequence of chemotherapy administered in ALL is dysfunction of the immune system, which persists during remission and after the end of the therapy [9]. In particular, T-cell-mediated cellular immune response can be affected by the chemotherapy, leading to the impairment of the immune system. In this study we aimed to investigate in detail different T-cell subpopulations in the peripheral blood of children following completed therapy for ALL in comparison with a group of healthy children.

## 2. Results

The used antibody panel made it possible to identify 52 subpopulations of T cells. The absolute values of individual subpopulations are gathered in Table 1, Table 2, Table 3, Table 4, Table 5 and Table 6.

The study exhibited significant differences in the absolute leukocyte count, as well as in numbers of lymphocytes and granulocytes in PB between the study and control group. In the PB of children cured of ALL, significantly lower counts of leukocytes and granulocytes were observed, as compared to the healthy children, while the total lymphocyte count was significantly higher in comparison to the control group (Table 1).

Significant differences were also found between the major T-cell subpopulation counts; the absolute values of double-positive CD4+CD8+ (*p* < 0.01), CD8+ (*p* < 0.05) and TCRγδ+ T cells (*p* < 0.01) were higher in the study group, whereas the value of the CD4/CD8 ratio within the T cells was lower, as compared to the control (*p* < 0.05; Table 2).

Furthermore, we observed significantly higher absolute numbers of three memory T-cell subpopulations in the study group as compared to the control group, comprising the total CD45RO+ T cells, CD45RO+CD8+ T cells, and CD45RO+CD27+ T cells (*p* < 0.01). In contrast, no differences were observed for CD45RO+CD4+, CD45RO+CD28+ memory T cells, nor for all CD45RA+ naïve T cells between the study and control groups (Table 3).

Among the activated T cells, significantly higher absolute counts of the HLA-DR+, HLA-DR+CD8 (*p* < 0.01), HLA-DR+CD57+, and CD25+CD8+ (*p* < 0.05) subpopulations were observed in the study group vs. the control group. Conversely, lower absolute values of CD69+ T cells were observed in the study group as compared to the healthy children. We also did not observe significant differences in the absolute count of regulatory T cells (Table 4) and almost all subpopulations of cytotoxic T cells between the two groups, except for the number CD57+ T cells, which was significantly higher in children cured of ALL as compared to the control group (*p* < 0.01; Table 5).

When it comes to the expression of chemokine receptors, significantly higher absolute values of the CCR7+, CCR7+CD45RO+, CCR7+CD28+, and CXCR4+CCR5+ T-cell subpopulations were observed in the children cured of ALL than in healthy children (Table 6).

## 3. Discussion

Treatment of ALL involves the use of chemotherapy, which on the one hand guarantees the elimination of leukemic blast cells, but on the other hand may negatively affect normal lymphocytes leading to the impairment of immune system function. In our study we aimed to assess to what extent the chemotherapy administered during the treatment of children with ALL influences the composition of immune cells, with particular focus on different T-cell subpopulations. Of the 52 T-cell subpopulations analyzed, 16 (30.8%) exhibited statistically significant differences between the study and control groups. Higher values in children after ALL treatment compared to healthy children were observed in 4 out of 6 major T-cell subpopulations, 3 out of 5 memory T-cell subpopulations, 4 out of 7 activated T-cell subpopulations, 1 out of 14 cytotoxic T-cell subpopulations, and 4 out of 17 T-cell subpopulations classified by chemokine receptors. Lower values in children after ALL treatment compared to healthy children were found in only one out of seven activated T-cell subpopulations.

According to Salem et al., a steady decrease in both CD4+ and CD8+ T-cell numbers takes place during chemotherapy, thus maintaining a constant CD4/CD8 ratio within the reference range [10]. However, our results show that, in children during the recovery process after ALL treatment, this value was lower compared to the control group (1.21 vs. 1.47, respectively, *p* = 0.02). This indicates a faster and more intensive recovery rate for cytotoxic CD8+ T-cell subpopulations, which are mainly involved in the elimination of virus-infected cells and abnormal cells, including cancer cells [11]. On the other hand, this result may also indicate the slower recovery rate of CD4+ helper T cells, which play a role in B-cell activation, or in enhancing the activity of CD8+ T cells and macrophages [11,12]. Similar results were obtained by the Mackall et al. group. This mechanism is most likely related to various extrathymic mechanisms of the maturation of CD4+ and CD8+ T cells [13].

We also observed higher absolute numbers of TCRγδ+ T cells in children cured of ALL, as compared to the healthy children. This observation may result from using therapeutic agents that indirectly activate this subpopulation, such as aminobisphosphonates, which are used in treatment of ALL [14]. This may also reflect the fact that TCRγδ+ T cells participate in the elimination of cancerous cells, similarly to NK cells and CD8+ T cells [14,15].

It was shown by van Tilburg et al. that naïve T-cell regeneration can be rapid after treatment completion, and can return to normal even within a month. In contrast, memory T-cell regeneration is much slower and can take up to 5 years [16]. However, our results did not support this observation, as the absolute count of memory T cells was significantly higher than in healthy control (1.03 (0.88–1.11) [G/l] vs. 0.78 (0.64–0.90) [G/l], respectively, *p* = 0.003), suggesting that this particular fraction of T cells was not severely affected by the ALL therapy. Similar results were obtained by Haining et al., who suggested that an increased number of memory T cells reduces the risk of death that may result from chemotherapy-related infections [17].

The increased level of activated T cells in children cured of ALL, as compared to the healthy children, may have many overlapping causes. After ALL treatment completion, patients are at risk of the reactivation of latent viral infections; e.g., Epstein–Barr virus (EBV) or cytomegalovirus (CMV). The continuous response to these antigens can cause T-cell activation [18,19]. Another mechanism of T-cell activation is chemotherapy-related tissue damage. The resulting inflammation is characterized by elevated levels of proinflammatory cytokines (e.g., IL-1, IL-6, or IFN-γ), which drive T-cell activation. The chronic persistence of inflammation can lead to the continued activation of T cells [20,21]. The chemotherapy and long-term antibiotic therapy used in the treatment of ALL can also negatively influence the gut microbiota, causing dysbiosis and changes in species composition, which also can lead to chronic antigenic stimulation and the activation of T cells [22,23,24].

We also demonstrated higher absolute counts of CD57+ T cells in children during the ALL therapy recovery process in comparison to the control group. Similarly to the case of activated T cells, this may be related to chronic antigenic stimulation. CD57 is a marker of aging of lymphocytes and a of a prolonged state of activation rather than functional exhaustion [25]. Initially, the activation stimuli are related to long-term treatment and tissue damage. In turn, after the treatment, activation of the immune system can be sustained by exposure to EBV or CMV, which may explain the increase CD57+ T cells, as well as the total numbers of activated T cells [25,26].

The results obtained in our study also showed that children recovering from ALL therapy tend to have a clearly higher number of CCR7+ T cells than the control group. This marker, as a key protein involved in the migration of immune cells, is responsible for the accumulation of these cells in peripheral lymphatic organs, such as lymph nodes. It is required for proper antigen presentation and antigen response [27]. The relatively high counts of CCR7+ T cells in children cured of ALL can indicate the good efficiency of the thymus in terms of the generation of naive T cells and their subsequent transition into memory T cells. A relatively higher count of CCR7+ T cells may be a hallmark of the intensive rebuilding of the immune system [28,29].

The recovery of the immune system after ALL treatment is long and continues even for a year after treatment. Many alterations in T-cell subpopulations between children after ALL treatment and healthy children indicate the frequent immunization of patients, what may indicate exhaustion of the immune system. But there are also positive aspects to the rebuilding of T-cell subpopulations, such as a higher number of TCRγδ+ T cells, which are responsible for the elimination of cancer cells.

## 4. Materials and Methods

The study group consisted of PB samples collected from 20 patients, aged 5 to 18 years (mean 10.6 years), exactly one year after ALL treatment completion. Patients were treated according to the consecutive chemotherapy protocols utilized over the years by the Polish Paediatric Leukaemia and Lymphoma Study Group (PPLLSG); specifically BFM’90 for SRG and MRG, New York, and ALL-IC BFM 2002 for the SRG, IRG, and HRG groups [30,31,32]. The control group comprised PB samples from 50 healthy children, aged 3.5 to 18 years (mean 12.6 years). All samples were drawn into heparinized tubes, and the staining was performed on the same day, within 4 h of collection.

Immunophenotyping of the analyzed cells was performed using a 6-color, 11-tube panel of monoclonal antibodies (Becton Dickinson, San Jose, CA, USA, Dako, Glostrup, Denmark, Beckman Coulter Brea, CA, USA, Biorad AbD Serotec, Kidlington, UK), as detailed in Table 7. Sample preparation and staining procedures were performed according to the EuroFlow consortium protocols [33]. Stained PB samples were analyzed using a FACS Canto II flow cytometer (Becton Dickinson). Example gating of the population is shown in Figures S1 and S2. Due to the non-normal distribution of the data, the absolute values of individual T-cell subpopulations were compared between the study and control groups using the Mann–Whitney U-test, with *p*-values < 0.05 considered statistically significant; however, the correction for multiple testing was not performed. We acknowledge that the lack of multiple testing correction, as well as the imbalance between the study and the control group constitute methodological limitations of our study.

## Figures and Tables

**Table 1:** Absolute values of the major leukocyte subpopulations in the peripheral blood of children.

Population	Study Group [G/l] Median (IQR)	Control Group [G/l] Median (IQR)	*p*
Leukocytes	5.87 (3.85–8.03)	7.45 (4.86–10.09)	<0.05
Total Lymphocytes	3.48 (1.54–5.42)	2.20 (1.74–2.62)	0.004
B cells	0.55 (0.35–0.71)	0.37 (0.30–0.48)	0.2
T cells	2.45 (2.15–2.59)	1.87 (1.73–2.02)	0.24
Granulocytes	3.52 (0.85–6.19)	3.89 (3.08–4.33)	0.003
Monocytes	0.72 (0.63–0.96)	0.58 (0.40–0.70)	0.09

**Table 2:** Absolute values of the main T-cell subpopulations in the peripheral blood of children cured of ALL and the healthy control group.

Population	Study Group [G/l] Median (IQR)	Control Group [G/l] Median (IQR)	*p*
TCRαβ+ T cells	1.80 (1.75–1.84)	1.69 (1.61–1.71)	0.43
TCRγδ+ T cells	0.23 (0.19–0.29)	0.11 (0.08–0.18)	0.002
CD4+ T cells	0.98 (0.90–1.17)	0.97 (0.89–1.11)	0.35
CD8+ T cells	0.84 (0.57–0.90)	0.63 (0.55–0.72)	0.04
CD4+CD8+ T cells	0.22 (0.06–0.39)	0.14 (0.03–0.24)	0.001
CD4+/CD8+ ratio	1.21 (1.04–2.06)	1.47 (1.22–1.97)	0.02

**Table 3:** Absolute values of naive and memory T cells in peripheral blood of children cured of ALL and healthy control group.

Population	Study Group [G/l] Median (IQR)	Control Group [G/l] Median (IQR)	*p*
Naïve T cells			
CD45RA+ T cells	0.19 (1.08–1.30)	1.10 (0.97–1.21)	0.28
CD45RA+CD4+ T cells	0.55 (0.46–0.68)	0.56 (0.45–0.64)	0.51
CD45RA+CD8+ T cells	0.52 (0.41–0.64)	0.43 (0.35–0.53)	0.06
CD45RA+CD27+ T cells	1.06 (0.90–1.20)	1.03 (0.84–1.17)	0.25
Memory T cells			
CD45RO+ T cells	1.03 (0.88–1.11)	0.78 (0.64–0.90)	0.003
CD45RO+CD4+ T cells	0.51 (0.41–0.67)	0.49 (0.39–0.58)	0.06
CD45RO+CD8+ T cells	0.29 (0.25–0.40)	0.20 (0.16–0.26)	0.0006
CD45RO+CD27+ T cells	0.85 (0.72–1.04)	0.67 (0.57–0.76)	0.003
CD45RO+CD28+ T cells	0.57 (0.43–0.74)	0.51 (0.46–0.63)	0.57

**Table 4:** Absolute values of T-cell activation markers and regulatory T cells in peripheral blood of children cured of ALL and healthy control group.

Population	Study [G/l] Mean ± SD	Control group [G/l] Mean ± SD	*p*
Activated T cells			
CD25+ T cells	0.17 ± 0.08	0.15 ± 0.09	0.16
CD25+CD8+ T cells	0.04 ± 0.04	0.04 ± 0.08	0.03
HLA-DR+ T cells	0.32 ± 0.19	0.18 ± 0.11	0.00003
HLA-DR+CD8+ T cells	0.18 ± 0.15	0.1 ± 0.09	0.001
CD69+ T cells	0.1 ± 0.1	0.15 ± 0.09	0.001
HLA-DR+CD69+ T cells	0.04 ± 0.04	0.03 ± 0.04	0.09
HLA-DR+CD57+ T cells	0.09 ± 0.07	0.04 ± 0.04	0.01
Regulatory T cells			
CD25+CD4+ T cells	0.12 ± 0.06	0.11 ± 0.05	0.68

**Table 5:** Absolute numbers of T cells expressing cytotoxic markers in peripheral blood of children cured of ALL and healthy control group.

Population	Study Group [G/l] Median (IQR)	Control Group [G/l] Median (IQR)	*p*
Vα24+ T cells	0.01 (0.008–0.013)	0.01 (0.01–0.01)	0.38
Vα24+CD8+ T cells	0.004 (0.02–0.004)	0.002 (0.001–0.003)	0.04
Vα24+CD4+ T cells	0.01 (0.005–0.01)	0.01 (0.004–0.01)	0.47
Vβ11+ T cells	0.02 (0.01–0.02)	0.01 (0.01–0.02)	0.1
Vβ11+CD8+ T cells	0.006 (0.003–0.01)	0.01 (0.004–0.01)	0.16
Vβ11+CD4+ T cells	0.01 (0.01–0.01)	0.01 (0.01–0.01)	0.29
Perforin+ T cells	0.02 (0.01–0.06)	0.05 (0.01–0.12)	0.26
Perforin+CD8+ T cells	0.02 (0.003–0.04)	0.03 (0.01–0.07)	0.5
CD56+ T cells	0.04 (0.02–0.07)	0.05 (0.03–0.07)	0.41
CD56+CD8+ T cells	0.02 (0.01–0.03)	0.03 (0.01–0.05)	0.23
CD16+ T cells	0.02 (0.01–0.03)	0.01 (0.01–0.03)	0.83
CD16+CD8+ T cells	0.01 (0.01–0.01)	0.01 (0.003–0.01)	0.43
CD94+ T cells	0.14 (0.12–0.20)	0.10 (0.08–0.13)	0.79
CD57+ T cells	0.22 (0.16–0.31)	0.15 (0.13–0.20)	0.001

**Table 6:** Absolute numbers T cells expressing chemokine receptors in peripheral blood of children cured of ALL and healthy control group.

Population	Study Group [G/l] Median (IQR)	Control Group [G/l] Median (IQR)	*p*
CCR7+ T cells	0.59 (0.49–0.83)	0.06 (0.03–0.24)	<0.05
CCR7+CD45RO+ T cells	0.10 (0.07–0.13)	0.02 (0.02–0.01)	0.001
CCR7+CD28+ T cells	0.59 (0.49–0.82)	0.05 (0.03–0.24)	<0.05
CXCR4+ T cells	0.78 (0.62–0.96)	0.38 0.25–0.72)	0.12
CXCR4+CD4+ T cells	0.25 (0.16–0.41)	0.13 (0.09–0.25)	0.32
CXCR4+CD8+ T cells	0.42 (0.34–0.66)	0.19 (0.10–0.38)	0.01
CXCR4+CCR5+ T cells	0.10 (0.03–0.20)	0.07 (0.01–0.13)	0.62
CCR5+ T cells	0.34 (0.22–0.45)	0.33 (0.21–0.47)	0.27
CCR5+CD8+ T cells	0.16 (0.10–0.21)	0.16 (0.11–0.29)	0.22
CCR5+CD4+ T cells	0.07 (0.04–0.12)	0.08 (0.05–0.14)	0.16
CCR4+ T cells	0.23 (0.21–0.29)	0.18 (0.11–0.31)	0.13
CCR4+CD8+ T cells	0.06 (0.03–0.11)	0.03 (0.01–0.08)	0.05
CCR4+CD4+ T cells	0.17 (0.15–0.21)	0.15 (0.10–0.18)	0.06
CXCR3+ T cells	0.98 (0.93–1.13)	0.81 (0.73–0.92)	0.1
CXCR3+CD8+ T cells	0.55 (0.47–0.64)	0.43 (0.37–0.51)	0.36
CXCR3+CCR5+ T cells	0.27 (0.17–0.36)	0.24 (0.17–0.32)	0.5
CXCR3+CXCR4+ T cells	0.39 (0.30–0.54)	0.17 (0.08–0.32)	0.04

**Table 7:** Antibody panel used for multiparametric immunophenotyping of peripheral blood.

	FITC	PE	PerCPCy5.5	PE-Cy7	APC	APC-Cy7
1	CD3	CD16+CD56	CD45	--	CD19	--
2	CD15	CD13	CD45	CD16	CD11b	CD14
3	CD2	CD56	CD3	CD7	CD5	CD8
4	CD27	CD45RA	CD3	CD4	CD45RO	CD8
5	CD28	CCR7	CD3	CD8	CD45RO	CD27
6	TCRαβ	TCRγδ	CD3	CD8	CD25	CD4
7	CD94	CD16	CD3	CD56	CD8	CD2
8	CD57	Perforin	CD3	CD16	HLA-DR	CD8
9	CD57	CD38	CD3	CD69	HLA-DR	CD8
10	Vα24	Vβ11	CD3	CD56	CD4	CD8
11	CD4	CXCR3	CD3	CCR5	CXCR4	CD8

## Data Availability

Requests to access the datasets should be directed to the corresponding author. Some raw flow cytometric data are not available due to existing data storage time limitations.

## Supplementary Materials

The following supporting information can be downloaded at: https://www.mdpi.com/article/10.3390/ijms262211107/s1.

## Abbreviations

The following abbreviations are used in this manuscript:

ALL	acute lymphoblastic leukemia
BM	bone marrow
CMV	cytomegalovirus
EBV	Epstein–Barr virus
PB	peripheral blood
