# Correction: Perkowski et al. Regeneration of Peripheral Blood T-Cell Subpopulations in Children After Completion of Acute Lymphoblastic Leukemia Treatment. *Int. J. Mol. Sci.* 2025, *26*, 11107

**DOI:** 10.3390/ijms27209317

**Published:** 2026-10-20

**Authors:** Bartosz Perkowski, Łukasz Słota, Aleksandra Lasia, Tomasz Szczepański, Łukasz Sędek

**Affiliations:** 1 Department of Pediatric Hematology and Oncology, Medical University of Silesia in Katowice, ul. 3 Maja 13-15, 41-800 Zabrze, Poland; bartosz.perkowski@sum.edu.pl (B.P.); lslota@sum.edu.pl (Ł.S.); aleksandra.lasia@sum.edu.pl (A.L.); tszczepanski@sum.edu.pl (T.S.); 2 Department of Microbiology and Immunology, Medical University of Silesia in Katowice, ul. Jordana 19, 41-808 Zabrze, Poland

In the original publication [1], there was a mistake in Section 2. Results, Paragraph 3: “Significant differences were also found between the major T-cell subpopulation counts; the absolute values of double-positive CD4+CD8+ (*p* < 0.01) and CD8+ (*p* < 0.05) T cells were higher in the study group, whereas the value of TCRγδ+ T cells (*p* < 0.01) and the CD4/CD8 ratio within the T cells were lower, as compared to the control (*p* < 0.05; Table 2).” The corrected sentence should be “Significant differences were also found between the major T-cell subpopulation counts; the absolute values of double-positive CD4+CD8+ (*p* < 0.01), CD8+ (*p* < 0.05) and TCRγδ+ T cells (*p* < 0.01) were higher in the study group, whereas the value of the CD4/CD8 ratio within the T cells was lower, as compared to the control (*p* < 0.05; Table 2).”

The same mistake occurs in the Abstract. The sentence “Children after ALL treatment had lower absolute values of TCRγδ+ and higher values of double-positive CD4+CD8+ and CD8+ T Cells.” should be corrected to “Children after ALL treatment had higher absolute values of TCRγδ+, double-positive CD4+CD8+ and CD8+ T Cells.”

The authors state that the scientific conclusions are unaffected. This correction was approved by the Academic Editor. The original publication has also been updated.

## References

[1] Perkowski B., Słota Ł., Lasia A., Szczepański T., Sędek Ł. (2025). Regeneration of Peripheral Blood T-Cell Subpopulations in Children After Completion of Acute Lymphoblastic Leukemia Treatment. Int. J. Mol. Sci..

