# Supplementary material for: Regeneration of Peripheral Blood T-Cell Subpopulations in Children After Completion of Acute Lymphoblastic Leukemia Treatment
Source: Int J Mol Sci. 2025 Nov 17;26(22):11107. doi: 10.3390/ijms262211107 (PMC12652639; doi:10.3390/ijms262211107)
Supplement: Supplementary file 1 [file ijms-26-11107-s001.zip › ijms-3870340-supplementary.pdf]

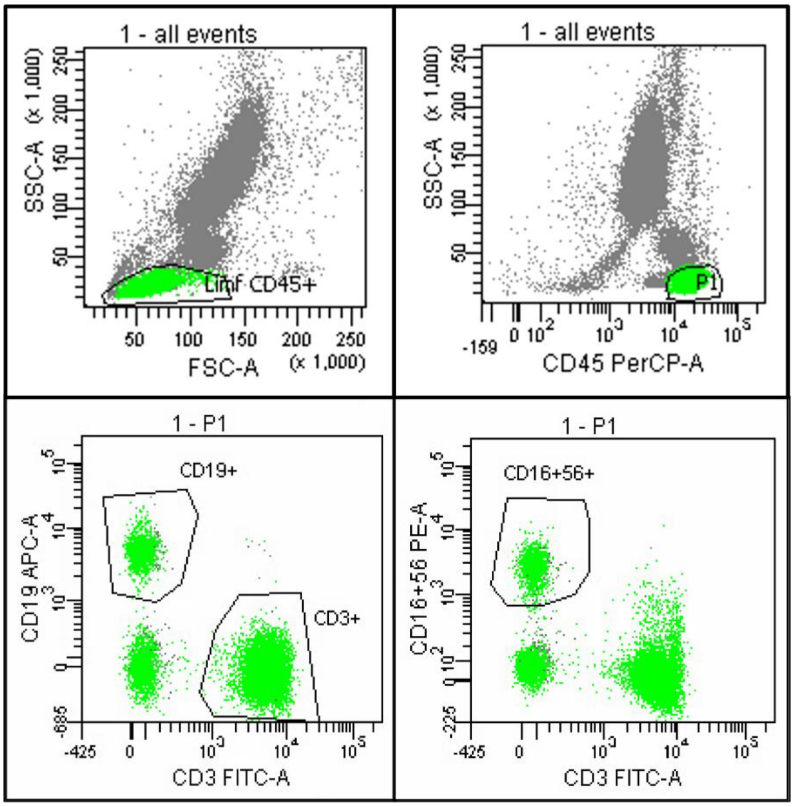

Figure S1. Representative flow cytometry gating strategy for primary lymphocyte populations: T-cells, B-cells and NK-cells.

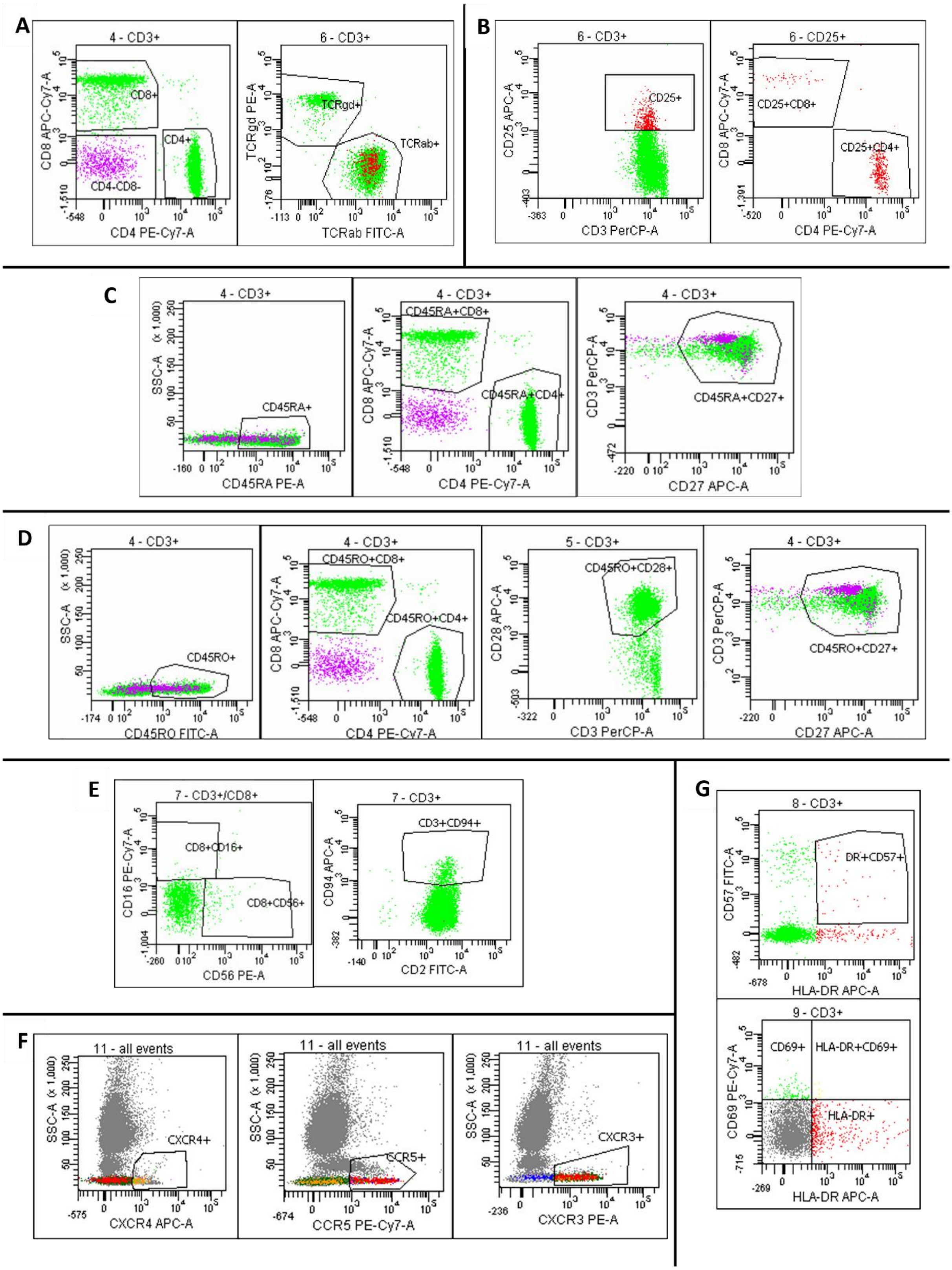

Figure S2. Selected key T-cell subpopulations. A – dissection of T-cells into CD4+/CD8+ and TCRαβ+/TCRγδ+ subpopulation; B – regulatory T-cells; C – naïve T-cells; D – memory T-cells; E – cytotoxic T-cells; F – chemokine receptor-positive T-cell subpopulations; G –activated T-cells.
